# From Platform Development to Scale-Up: Stable Producer Cell Line-Based Lentiviral Vector Manufacturing

**DOI:** 10.3390/pharmaceutics18101265

**Published:** 2026-10-06

**Authors:** Ane Arrasate, Igone Bravo, Haizea Fernandez, Carolina Amagliani, Carlos Salgado, Maria Garcia, Carolina Guiance, Maite Azcarate, Tania Arias, Carlos Lopez-Robles, Maria Carazo-Hidalgo, Lorea Blazquez, Cristina Garcia, Maria Echeverria, Andrés Lamsfus-Calle, Marie J. Fertin

**Affiliations:** 1 VIVEbiotech, 20014 Donostia-San Sebastián, Spain; ibravo@vivebiotech.com (I.B.); hfernandez@vivebiotech.com (H.F.); mgarcia@vivebiotech.com (M.G.); cguiance@vivebiotech.com (C.G.); mazcarate@vivebiotech.com (M.A.); tarias@vivebiotech.com (T.A.); clopez@vivebiotech.com (C.L.-R.); cgarcia@vivebiotech.com (C.G.); mecheverria@vivebiotech.com (M.E.); alamsfus@vivebiotech.com (A.L.-C.); mfertin@vivebiotech.com (M.J.F.); 2 Department of Biochemistry and Molecular Biology, University of the Basque Country UPV/EHU, 48940 Bilbao, Spain; 3 Neurogenetics, RNA Biology and Therapies Group, Department of Neurosciences, Biogipuzkoa Health Research Institute, 20014 Donostia-San Sebastián, Spain; maria.carazohidalgo@bio-gipuzkoa.eus (M.C.-H.); lorea.blazquezgarcia@bio-gipuzkoa.eus (L.B.); 4 Centro de Investigación Biomédica en Red de Enfermedades Neurodegenerativas (CIBERNED), Instituto de Salud Carlos III, 28029 Madrid, Spain; 5 Ikerbasque, Basque Foundation for Science, 48009 Bilbao, Spain

**Keywords:** lentiviral vectors, gene therapy, stable producer cell line, lentiviral packaging cell line, cell line development, cell line scale up, adherence, bioreactor, SB100X, transposase

## Abstract

**Background**: The development of stable producer cell lines (SCLs) represents a promising alternative for lentiviral vector (LVV) manufacturing, offering improved reproducibility and reduced manufacturing costs. In previous work, our group established an SCL generation platform based on a well-characterized packaging cell line (PCL), demonstrating successful vector production at laboratory scale. However, translation of this platform to larger scales remains necessary for industrial applications. **Objective**: In this study, we aimed to evaluate the scalability of the existing platform and identify key parameters that influence the production performance (e.g., cell density, harvest timing or pH). Moreover, the platform was enhanced through extensive genetic characterization and improved integration methods. **Results**: Our findings suggest that mildly acidic culture conditions and extended harvest periods contribute to increased LVV titers. Furthermore, we conducted a comparative analysis of transgene integration strategies to determine the most suitable method for SCL generation. The results demonstrate that transposase-mediated integration provides a precise and stable approach, minimizing concatemer formation and tandem rearrangements. **Conclusions**: Collectively, these findings provide evidence that our stable producer cell line constitutes a scalable, genetically characterized and consistent LVV production platform, supporting its potential for large-scale LVV manufacturing.

## 1. Introduction

Gene and cell therapy have been the main field of application for lentiviral vectors (LVVs), preferentially for ex vivo approaches. Recently, their use has expanded rapidly into additional application areas, including in vivo approaches, gene editing and vaccine development, where they act as versatile multifunctional tools [1]. The outcome of the resulting therapies strongly relies on the LVV manufacturing process and characterization. Accordingly, the demand for large-scale production of lentiviral vectors has significantly increased. In this context, the LVV-specialized industry is also growing as Contract Development and Manufacturing Organizations (CDMOs) that are required to constantly and rapidly adapt to the requirements of the drug developers and the regulatory agencies. Thus, implementation of robust, reproducible and scalable processes is essential to ensure cost-efficient long-term manufacturing campaigns.

The current standard method for large-scale manufacturing of GMP-grade LVV starts with the upstream processing (USP). In general terms, this phase consists of HEK293T cell line culture expansion and transfection for viral vector production. The USP ends with the viral supernatant harvesting, the same point where the downstream phase (DSP) starts. The bulk harvest undergoes purification, concentration and formulation in the specific matrix before undergoing final sterile filtration. The final product is then characterized through functional or physical titer assays, together with purity and potency assessments [1]. While this process adequately supports current market demands, further optimization is necessary to improve reproducibility and reduce overall manufacturing costs [2].

The standard LVV manufacturing through transient transfection is a suitable method for small-scale production, although it poses some challenges when scaling up. It is a costly procedure, mainly due to the expensive raw materials, that allow limited control and reproducibility when transitioning into industrial-scale production [3,4,5]. One of the main strategies currently explored in the field is the development of LVV packaging cell lines (PCLs) or stable producer cell lines (SCLs), where viral helper genes are integrated in the cell genome, with SCLs additionally incorporating the transgene of interest [6,7,8]. These cell lines are mainly inducible [6,7,9,10] due to the potential toxicity of the LVV helper gene expression within the cell [11,12]. This technology represents a transfection-free LVV production that is controlled by adding or removing an inducer or repressor molecule. Although the generation of packaging and stable producer cell lines is arduous to accomplish, multiple benefits are achieved, particularly a reinforced reproducibility and a reduction in costs associated with plasmid and transfection reagents.

Previously, we developed a clinically relevant platform for the generation of SCLs based on a well-characterized and stable PCL [6]. The proof-of-concept SCL models exhibited promising LVV yields, with suitable recoveries in preliminary AEX purification experiments. However, all data were obtained under laboratory-scale conditions. For industrial applications, scaling up our SCL-based production from the laboratory to a bioreactor setting is a requirement. Therefore, in this study, we discussed the results of the SCL-based LVV production in fixed-bed bioreactor, including vector titer and purity data, which have enabled us to identify potential key factors for improving cell performance during scale-up. Additionally, we emphasized the importance of selecting an appropriate gene integration method and implementing robust clone screening strategies to ensure therapeutic transgene integrity and enhanced viral titer.

## 2. Materials and Methods

### 2.1. Cell Culture and Media

HEK293T cells (ATCC CRL-3216), PCLs and SCLs were maintained in Dubelcco’s modified Eagle’s medium with high glucose supplemented with Glutamax (DMEM high glucose from Fisher Scientific, Pittsburg, PA, USA), 10% fetal bovine serum (FBS, Merck, Darmstadt, Germany), 100 IU/mL penicillin and 100 μg/mL streptomycin (penicillin-streptomycin solution, Merck, Darmstdat, Germany). The constructs used for the generation of the PCL and SCL cell lines were described on our previous work [6].

Population doubling time of the cell culture is calculated with the following formulas:

(1) $$\mathrm{PDT}=\frac{\ln(2)}{\mathrm{Growth}\;\mathrm{rate}}$$

(2) $$\mathrm{Growth}\;\mathrm{rate}=\frac{\ln\left(\frac{\mathrm{final}\;\mathrm{nº}\;\mathrm{cells}}{\mathrm{initial}\;\mathrm{nº}\;\mathrm{cells}}\right)}{\mathrm{time}\;(\mathrm{hours})}$$

### 2.2. Large-Scale LVV Production

SCL was seeded at a 12,000 cell/cm^2^ density in a 2.4 m^2^ Scale-X™ hydro system (Univercells Technologies, Nivelles, Belgium) staged in an iCellis Nano Bioreactor controller (Cytiva, Marlborough, MA, USA). Culture parameters, including 40% dissolved oxygen (DO), 5% CO_2_, 90% humidity, temperature (36.5 °C) and 7–7.2 pH were continuously monitored in-line and controlled. Cell growth was indirectly assessed by off-line glucose consumption measurement with the Bioprofile^®^ FLEX2 (Nova biomedical, Barcelona, Spain). When the aimed glucose concentration was reached (1.5–2.5 g/L), corresponding to the lower and upper limits of the glucose range known to support active cellular metabolism, cultures were induced with 10 ng/mL doxycycline hydrochloride (Sigma-Aldrich, St. Louis, MO, USA) and maintained under perfusion. Production was carried out in perfusion mode at a 5 L working volume, maintaining a system hold-up volume of 800 mL. Viral supernatants were harvested every 20 h and subsequently and pooled into a single bulk harvest 60 h post-induction.

### 2.3. Small-Scale LVV Production

In routine experiments, SCL was seeded in a multiwell-6 culture plate (Corning, Tewskbuy, MA, USA) and 18 h later, when cell confluency was 70–80%, production was induced with 1 ng/mL of doxycycline hydrochloride. Cells were maintained at 37 °C in a humidified incubator (90% humidity) under an atmosphere of 5% CO_2_ and 95% DO. Viral supernatant (VSN) was harvested 60–72 h after induction and filtered using a 0.45 μm filter (Millex-HA, Merck, Darmstadt, Germany) to later aliquot and freeze at −80 °C until further analyses.

When multiwell-6 culture plate production was performed as a downscale model of bioreactor production, SCL was seeded at a 12,000 cell/cm^2^ density in a working volume of 2 mL per well. Cell growth was indirectly measured by quantifying glucose consumption through the Bioprofile^®^ FLEX2 (Nova biomedical, Barcelona, Spain). Consequently, cell induction was performed at a determined glucose concentration with 1 ng/mL doxycycline hydrochloride, corresponding to the same doxycycline-to-cell ratio used in large-scale production. The following procedure for incubation and harvesting remained identical to the aforementioned small-scale production.

### 2.4. Downstream Processing (DSP) of LVV

Large-scale LVV production batches were purified and concentrated through multi-step DSP workflow. The bulk harvest was initially clarified through a 0.45 μm filter (Cytiva, Marlborough, MA, USA), treated with Denarase^®^ endonuclease (C-LEcta, Leipzig, Germany) for nucleic acid digestion, and conditioned for anion-exchange chromatography (AEX). Purification was performed using an AKTA pure 25™ system (Cytiva, Marlborough, MA, USA) equipped with Mustang™ Q XT membrane capsules (Cytiva, Marlborough, MA, USA). The eluted material was further filtered (0.2 μm), concentrated and buffer-exchanged by tangential flow filtration (TFF) with a 300 kDa Hydrosart membrane (Sartorius AG, Göttingen, Germany), followed by final sterile filtration through a 0.2 μm PES filter (Cytiva, Marlborough, MA, USA).

### 2.5. LVV Infective Titer Calculation

Functional LVV titers were determined by transduction of HEK293T cells with serial dilutions of viral supernatants in the presence of 8 μg/mL of Polybrene^®^ (Santa Cruz Biotechnology Inc., Dallas, TX, USA). Transduced cells were cultured for 72 h, after which transduction units were measured by flow cytometry (CytoFLEX, Beckman Coulter, Brea, CA, USA) based on GFP expression, and viral titers were calculated accordingly. Alternatively, transduction units were similarly quantified assessing the viral copy number integrated into the transduced cell genome by quantitative PCR (qPCR). Genomic DNA was extracted with QIAamp DNA Mini Kit (Qiagen, Hilden, Germany) and analyzed by qPCR utilizing SYBR Green Master Mix (ThermoFisher Scientific, Waltham, MA, USA) and the QuantStudio^TM^ 3 thermocycler (ThermoFisher Scientific, Waltham, MA, USA). Retrotranscribed proviral DNA was amplified with specific primers, enabling the determination of transducing units per cell (TU/cell) and calculation of viral titer.

### 2.6. ELISA for p24 Quantification

Viral particle concentration was determined by p24 protein measurement using an ELISA assay performed by the automated Gyrolab xPlore™ instrument and the kit Gyrolab Bioaffy 1000 HC Toolbox (Gyros Protein Technologies, Uppsala, Sweden). LVV total viral particles were calculated based on previous calculations of 2000 p24 molecules per virion [13]. As p24 quantification may include both particle-associated and free p24 protein present in the supernatant, the calculated value should be considered an estimate of viral particle concentration.

### 2.7. ELISA for Host Cell Protein (HCP) Quantification

Host cell proteins (HCPs) from producer cells were quantified using a HEK293 HCP ELISA kit from Cygnus Technologies (Leland, NC, USA), following the manufacturer’s instructions. Absorbance was measured with the Synergy H4 Hybrid Reader (Agilent Biotek, Santa Clara, CA, USA). HCP concentrations were calculated in ng/mL based on assay output and subsequently normalized to the infectious titer.

### 2.8. Residual DNA Quantification

Residual DNA concentration was measured through Quant-iT™ PicoGreen™ dsDNA assay kit (ThermoFisher Scientific, Waltham, MA, USA), following the manufacturer’s instructions. Fluorescence signal was detected with the Synergy H4 Hybrid Reader (Agilent Biotek, Santa Clara, CA, USA). DNA concentrations were calculated in ng/mL based on assay output and subsequently normalized to the infectious titer.

### 2.9. Retro-Transduction Assessment

For viral particle retro-transduction into the producer cell, its genomic DNA was extracted according to the kit’s manufacturer instructions (QIAamp DNA Mini Kit, Qiagen, Hilden, Germany). Subsequently, a quantitative PCR was performed utilizing SYBR Green Master Mix (ThermoFisher Scientific, Waltham, MA, USA) and the QuantStudioTM 3 thermocycler (ThermoFisher Scientific, Waltham, MA, USA). The primers specifically amplified retrotranscribed proviral DNA, which made it possible to determine the transducing events per cell (TU/cells).

### 2.10. Transgene Copy Number Assessment

Cell genomic DNA was extracted according to the kit’s manufacturer instructions. Subsequently, two quantitative PCR were performed utilizing SYBR Green Master Mix and the QuantStudioTM 3 thermocycler. On the one hand, retro-transduction frequency was assessed following the instructions described above. This value was subtracted to the total number of integrated copies, which were measured by amplifying a cassette-specific region. The resulting value indicated the transgene copy number.

### 2.11. Whole Genome Sequencing (WGS)

A whole genome sequencing of the PCL was externally performed by LongSeq Applications (Murcia, Spain) based on CRISPR-Cas-targeted enrichment combined with long-read sequencing through Nanopore technology (Oxford Nanopore Technologies, Oxford, UK). Results were delivered as raw data that required exhaustive bioinformatic analysis. Library preparation was performed using Oxford Nanopore Technologies protocols (SQK-LSK114), and sequencing was carried out on a PromethION platform (FLO-PRO114M). Raw reads extracted from the sequencing were initially filtered through quality control and preprocessing steps. Those filtered reads were aligned to custom references containing helper constructs using Minimap2 and following Oxford Nanopore specific parameters. Subsequently, reads mapping to the helper constructs with a mapping quality (MAPQ) > 10 were aligned to the GRCh38 primary assembly to identify the genomic regions flanking the integration regions. Due to the absence of an appropriate reference genome and the high sequence homology between the host genome and several regions of the integrated helper constructs, alignments were also manually inspected to reconstruct and verify integration events.

### 2.12. SB100X In Vitro Transcription (IVT)

SB100X mRNA was generated by in vitro transcription using HiScribe™ T7 High Yield RNA Synthesis Kit (New England Biolabs, Inc., Ipswich, MA, USA), following the manufacturer’s instructions. To enhance mRNA stability, a 3-0-Me-m7G(5)ppp(5′)G RNA Cap Structure Analog (New England Biolabs, Inc. MA, USA) was added. The transcribed mRNA was subsequently purified using the MEGAclear™ (ThermoFisher scientific Inc., Waltham, MA, USA).

### 2.13. Transgene Integration (Random and SB100X)

Cells were seeded in multi-well-6 culture plates (Corning, Tewskbuy, MA, USA), and 20 h later, when the cells were at 40–50% confluency, the specific transgene plasmid was transfected. Random integration of the transgene was performed by transfecting the specific transgene as a linearized plasmid. Polyethyleneimine PEIpro^®^ (Polyplus, Illkirch-Graffenstaden, France) was used as a transfection reagent with a 2:1 PEI:DNA (*w*/*w*). SB100X-mediated transgene integration was performed by co-transfecting the SB100X mRNA along with the transgene plasmid with the transfection reagent JetMessenger^®^ (Polyplus, Illkirch-Graffenstaden, France). DNA:SB100X ratio was 1:0.5 and the ratio between the oligonucleotide cargo (DNA and RNA) to JetMessenger^®^ was 1:2. Both methods transfected the same amount of DNA, in order to be comparable with each other. Three days later, 0.18 μg/mL of puromycin (Merck KGaA, Darmstadt, Germany) was added to the media for selection.

### 2.14. Subcloning

SCL subcloning was performed by serial dilution method [14] reaching a concentration of 0.25 cells per well of a 96-well plate (Corning, Tewskbuy, MA, USA). Following their growth by microscope visualization, cell populations derived from single cells were expanded for further analyses and cell bank generation.

### 2.15. Targeted Locus Amplification (TLA) Study

SB100X-mediated transgene integrations were characterized in the SCL through a targeted locus amplification (TLA) assay performed by Solvias (Utrecht, The Netherlands). Samples for this analysis were prepared following the indications of Solvias. Results allowed the identification of the integration loci of the transgene construct into the cell genome.

## 3. Results

In the following lines, the results of the experimental and analytical work carried out to evaluate the scale up of SCL-based LVV production are described. Additionally, PCL genome and potential alternative integration strategies are assessed for efficient and safe platform development.

### 3.1. Bioreactor Production

#### 3.1.1. Upstream Processing (USP)

The SCL-based LVV production process was scaled up to a fixed-bed bioreactor providing 2.4 m^2^ surface for cell growth. The SCL evaluated was a monoclonal producer cell line engineered to generate third-generation LVV encoding a GFP-CAR (NKG2D) fusion protein driven by a MNDU3 promoter (SCL-CAR) and pseudotyped with a VSV-G envelope. In parallel, a small-scale batch in multi-well plate was performed as a control under equal experimental conditions using the same producer cell line. The scale-up corresponded to a 2500-fold increase in cell growth surface area with proportional increases in culture volume and total cell number. LVV production was induced in two independent bioreactors by doxycycline supplementation at two different glucose concentrations, 2.5 g/L and 1.5 g/L, respectively. This experimental design aimed to evaluate the impact of cell density at induction time in lentiviral production, as well as to compare the production scales. SCL growth rate was comparable for both scale conditions despite a slight delay in bioreactor culture that postponed induction timings: in large-scale cultures, 2.5 g/L glucose was reached at 120 h (Figure S1a) and 1.5 g/L at 127 h (Figure S1b), whereas in small-scale cultures, these concentrations were reached at 96 and 120 h (Figure S1c), respectively. Metabolite concentration profiles were broadly similar in both bioreactors (Figure S1). However, the condition induced at 1.5 g/L glucose showed overall lower glucose levels and higher lactate accumulation during the production phase (Figure S1), consistent with the higher cell density reached before and after induction.

**Figure 1:**
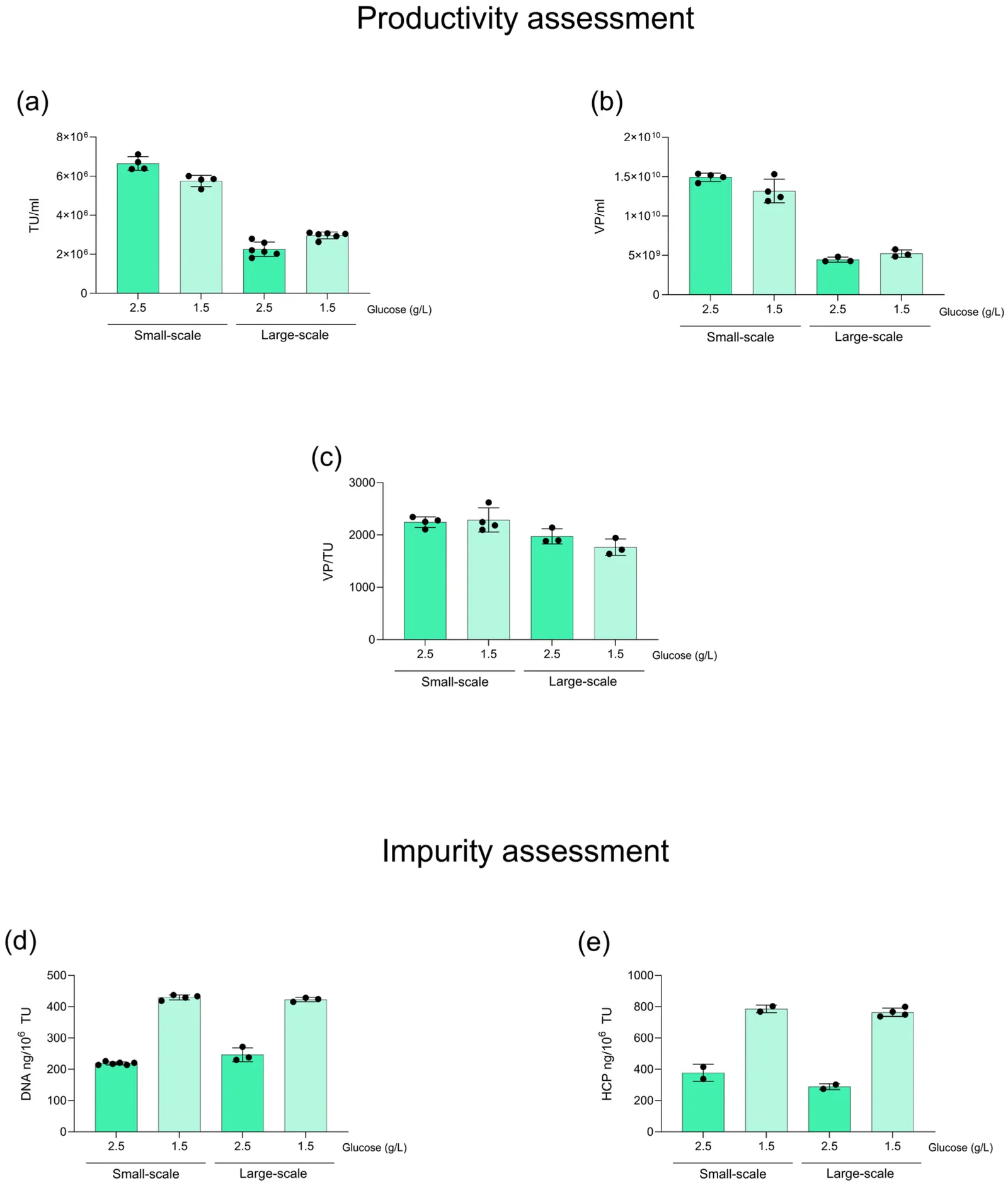
Comparison of LVV production across different scales and cell densities (2.5 and 1.5 g/L glucose levels). Results are shown in terms of (**a**) infectious titer as viral copy number per mL (TU/mL), (**b**) physical titer as viral particles per mL (VP/mL), (**c**) performance factor as ratio between viral particles and viral copy number (VP/TU). Additionally, impurity concentrations are also shown as (**d**) DNA concentration in ng per 1 × 10^6^ infectious viral particle (ng DNA/10^6^ TU). (**e**) HCP concentration in ng per 1 × 10^6^ infectious viral particle (ng HCP/10^6^ TU). Data are expressed as mean values, with error bars representing standard deviations, and with dots representing individual technical replicates.

The results of this experiment were evaluated in terms of the transferability of the newly developed SCL to scalable systems. Additionally, cell density, a key process parameter, was evaluated. In this line, the production process was assessed based on infective and physical titer. The relation of physical particles per infective ones, referred to as performance factor, was determined as an indicator of infectivity and quality of harvest product. These metrics were expressed per milliliter, given that the area-to-volume relationship was equivalent across both scales. Overall, the results show that the infective titer of the VSN was relatively higher when produced at a small-scale setup and that there were no substantial differences between cells induced at 2.5 g/L and 1.5 g/L of glucose (Figure 1a). At a small scale, the productions yielded (6.65 ± 0.35) × 10^6^ TU/mL (mean ± SD) at 2.5 g/L induction and (5.75 ± 0.29) × 10^6^ TU/mL at 1.5 g/L. In contrast, the corresponding values for the large-scale runs were (2.25 ± 0.36) × 10^6^ and (2.96 ± 0.17) × 10^6^ TU/mL, respectively. Physical particle production showed the same pattern, with small-scale runs yielding higher relative concentrations and both induction levels performing similarly (Figure 1b). Because both infectious and physical titers followed proportional trends, the ratio between these metrics remained comparable across all conditions and scales (Figure 1c), confirming that lentiviral particle infectivity was maintained independently of cell density at induction or scale.

Additionally, residual DNA and host cell protein (HCP) concentrations were measured as process-related impurities. The impurity concentration was normalized to the respective infectious titer so that the purity relative to the biologically active component is expressed. For DNA, approximately 200 ng per 1 × 10^6^ TU was detected in the 2.5 g/L condition, compared with roughly 400 ng in the 1.5 g/L condition (Figure 1d). Similarly, HCP levels per 1 × 10^6^ TU were roughly doubled in the 1.5 g/L culture compared to the 2.5 g/L condition (Figure 1e). Remarkably, the relative impurity levels were comparable between small- and large-scale productions, suggesting that the process-related impurities were consistent through bioreactor scale transfer. However, according to results, these levels were higher in 1.5 g/L glucose condition, likely reflecting the higher cell density resulting in increased cell-related impurities.

#### 3.1.2. Productivity Impact Assessment

The slightly reduced lentiviral productivity observed at the bioreactor scale in comparison with the small-scale system underscored the need to identify process parameters contributing to inter-scale variability. Among these factors, pH control of the culture medium was identified as a major difference between the two production setups. In contrast to the large-scale system, where pH is continuously controlled, the culture medium in small-scale experiments undergoes acidification due to cell growth and consequent lactate generation. Acidic pH has been strongly associated with increased productivity, due to prevention of VSV-G-mediated retro-transduction of producer cells [8,15,16,17]. To assess whether this factor contributed to the observed decrease in titer, a scale-down experiment was designed to mimic both operating conditions. In addition to pH evaluation, lentiviral supernatant harvesting time was also included in this experiment as an additional experimental variable. In contrast to transient transfection, where the transfection mixture is delivered as a single bolus, induction in a bioreactor is achieved by replacing the medium with doxycycline-containing medium. Under continuous perfusion, fresh medium supplemented with doxycycline is continuously supplied, ensuring a sustained and stable availability of the inducer. This difference in exposure dynamics likely contributes to the distinct production profiles observed. Whereas transient transfection-based production typically peaks around 48 h, SCL-based production continues to increase by up to 60 h. Therefore, we decided to assess pH control and extension of the production duration to assess their effect on the potential enhancement of LVV yields.

**Figure 2:**
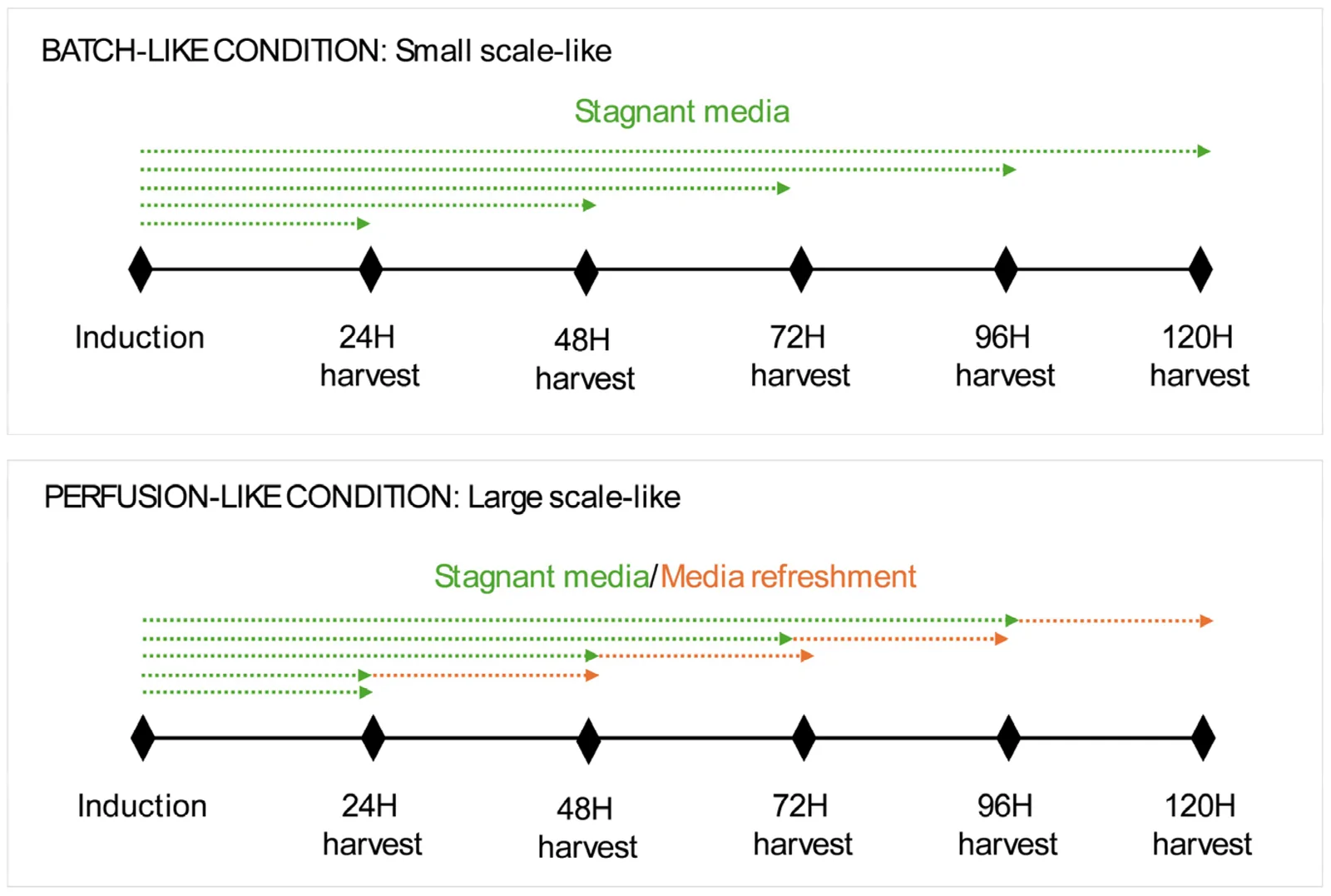
Schematic representation of the scale-down experiment performed to assess the productivity impact in the scale up. Dashed green arrows indicate the duration of supernatant accumulation in stagnant medium. Dashed orange arrows indicate the accumulation period after medium exchange in the perfusion-like condition.

To evaluate both variables, a small-scale experiment in 6-well plate format was performed. The VSN collection was performed for up to 120 h post-induction following two different harvesting strategies (Figure 2). On the one hand, a “batch-like” condition was replicated at small scale, where the medium is not replaced from the first induction until the specific harvesting time. Supernatants of the different replicates were collected at 24, 48, 72, 96 and 120 h after the initial induction. On the other hand, the “perfusion-like” condition aimed to mimic the controlled pH of large-scale production by collecting and refilling the wells with fresh doxycycline-supplemented medium at the previously specified time points. Harvested viral supernatants were analyzed in terms of titer and contaminants with the aim to determine the optimal harvesting time and to compare the harvesting models.

**Figure 3:**
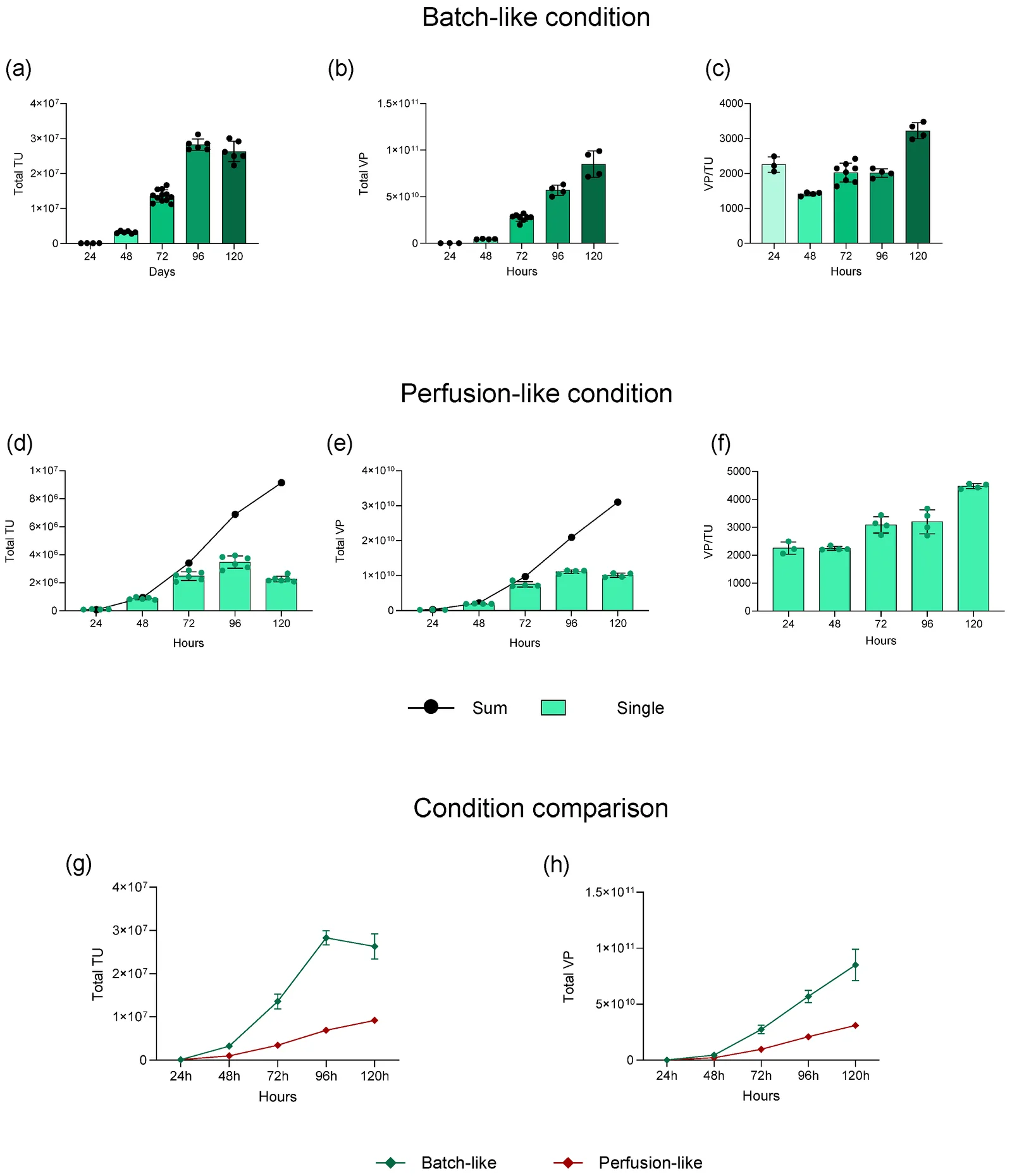
Comparison of LVV production under batch-like and perfusion-like conditions in a small-scale model. Batch-like condition total titer analysis as (**a**) infectious titer (TU), (**b**) physical titer (VP) and (**c**) performance factor (VP/TU). Perfusion-like condition total titer analysis as (**d**) infectious titer (TU), (**e**) physical titer (VP) and (**f**) performance factor (VP/TU). Columns represent the titer of each independent fraction and connected data-points show the sum of previous fractions. Data are expressed as mean values, with error bars representing standard deviations, and with dots representing individual technical replicates. Comparison of the cumulative production obtained under perfusion-like condition with the batch-like condition as (**g**) total TU and (**h**) total VP.

In the batch-like model, the yield of infectious LVV particles increased progressively over the first 96 h of production, after which it ceased to rise (Figure 3a). The viral supernatant reached an infective titer of (2.82 ± 0.16) × 10^7^ TU. The physical titer progressively increased even at 120 h, obtaining (8.51 ± 1.39) × 10^10^ VP (Figure 3b). Consequently, the performance factor was similar in all time points, although at 120 h it seemed slightly higher (Figure 3c), reflecting an increase in non-infectious particles relative to the infectious ones. As for the perfusion-like model, the titer of each fraction (columns) is displayed along with the overall total titer (dots), calculated as the sum of the corresponding fractions (Figure 3d,e). Regarding the infectious titer, the highest production time point was observed at 96 h (fraction titer of (3.47E ± 0.43) × 10^6^ TU), although continued production at 120 h still contributes to an increase in the total titer (sum is 9.23 × 10^6^ TU) (Figure 3d). Physical titer evaluation showed a similar pattern (Figure 3e), maintaining to some extent the performance factor in all the fractions, although the 120 h-fraction exhibited a higher value (Figure 3f). When comparing both production models, the infectious and physical titer obtained under batch-like condition exceeds that of the perfusion-like condition by more than two-fold (Figure 3g,h). Notably, at 96 h, offline measurements indicated that pH was 6.9 in the batch-like mode, whereas it remained at 7.1 in the perfusion-like mode. This data suggested that maintaining a constant pH through regular medium replacement might be compromising LVV production.

**Figure 4:**
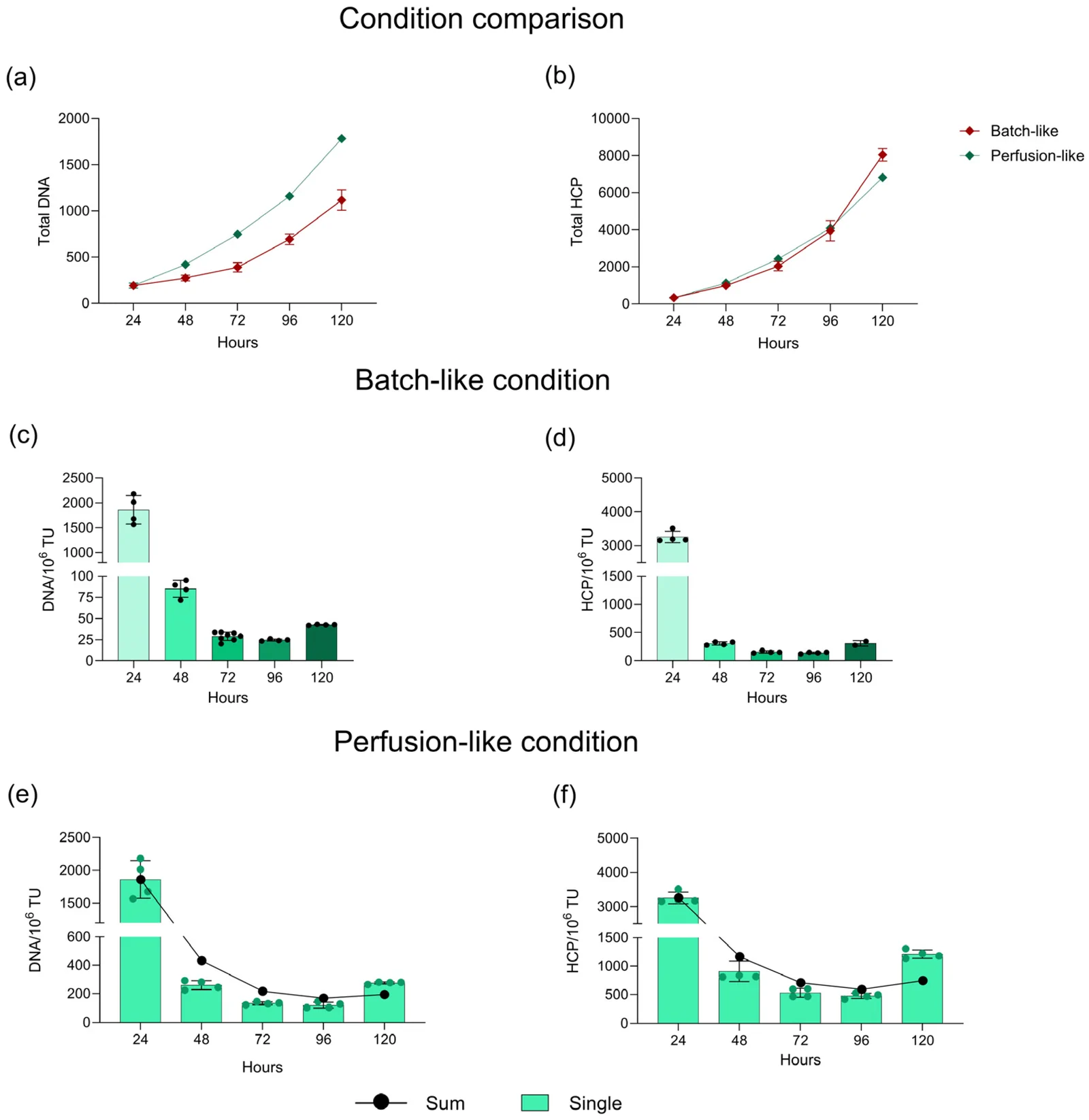
Comparison of LVV production under batch-like and perfusion-like conditions in a small-scale model. Impurity concentration in terms of (**a**) total DNA (ng) and (**b**) total HCP (ng). Impurity concentrations (ng) per 10^6^ TU for the batch-like model (**c**) DNA and (**d**) HCP, and for the perfusion-like model (**e**) DNA and (**f**) HCP. Columns represent the titer of each independent fraction and connected data-points show the sum of previous fractions. Data are expressed as mean values, with error bars representing standard deviations, and with dots representing individual technical replicates.

In terms of process-related impurities, host cell proteins and residual DNA concentrations were measured. Total DNA concentration (ng) in perfusion-like mode was two-fold higher than those in batch mode from day 2 onwards (Figure 4a). In contrast, HCP concentrations exhibited similar profiles under both conditions across all time points (Figure 4b). Normalizing contaminant levels to the corresponding infectious titers (expressed per 10^6^ infectious units) provided additional insight. In the batch-like model, both DNA and HCP per infectious particle decreased, reaching minimum values on day 4 (24.52 ng DNA/10^6^ TU and 139.16 ng HCP/10^6^ TU), with a slight increase observed on day 5 (Figure 4c,d). A comparable trend was observed under perfusion-like conditions: contaminant levels per infectious particle declined over time, with the lowest values also occurring on day 4 (166.42 ng DNA/10^6^ TU and 586.52 ng HCP/10^6^ TU) (Figure 4e,f).

Given that producer cell lines express the receptor for VSV-G-pseudotyped LVVs (LDLR), during lentiviral vector production, the continuous presence of LVV in the supernatant might lead to titer loss owing to virus re-entry or retro-transduction [17,18]. Thus, LVV re-entry frequency in the producer cells was evaluated for both models, batch- and perfusion-like. The results reveal a very different situation for each model (Figure S2). The batch-like model showed a very low re-entry frequency throughout the culture period (less than one copy per cell on average), with a slight peak at day 2 followed by a decline. This decrease may be attributed to different proliferation rates in retro-transduced and non-retro-transduced cells as the frequency represents an average of the whole population [19]. However, in perfusion-like conditions, the re-entry frequency increased over time, reaching more than 30 copies per cell on day 5. The increase in the re-entry might contribute to the observed decrease in overall infectious titer of the LVV yield in the supernatant.

#### 3.1.3. Downstream Processing (DSP)

**Figure 5:**
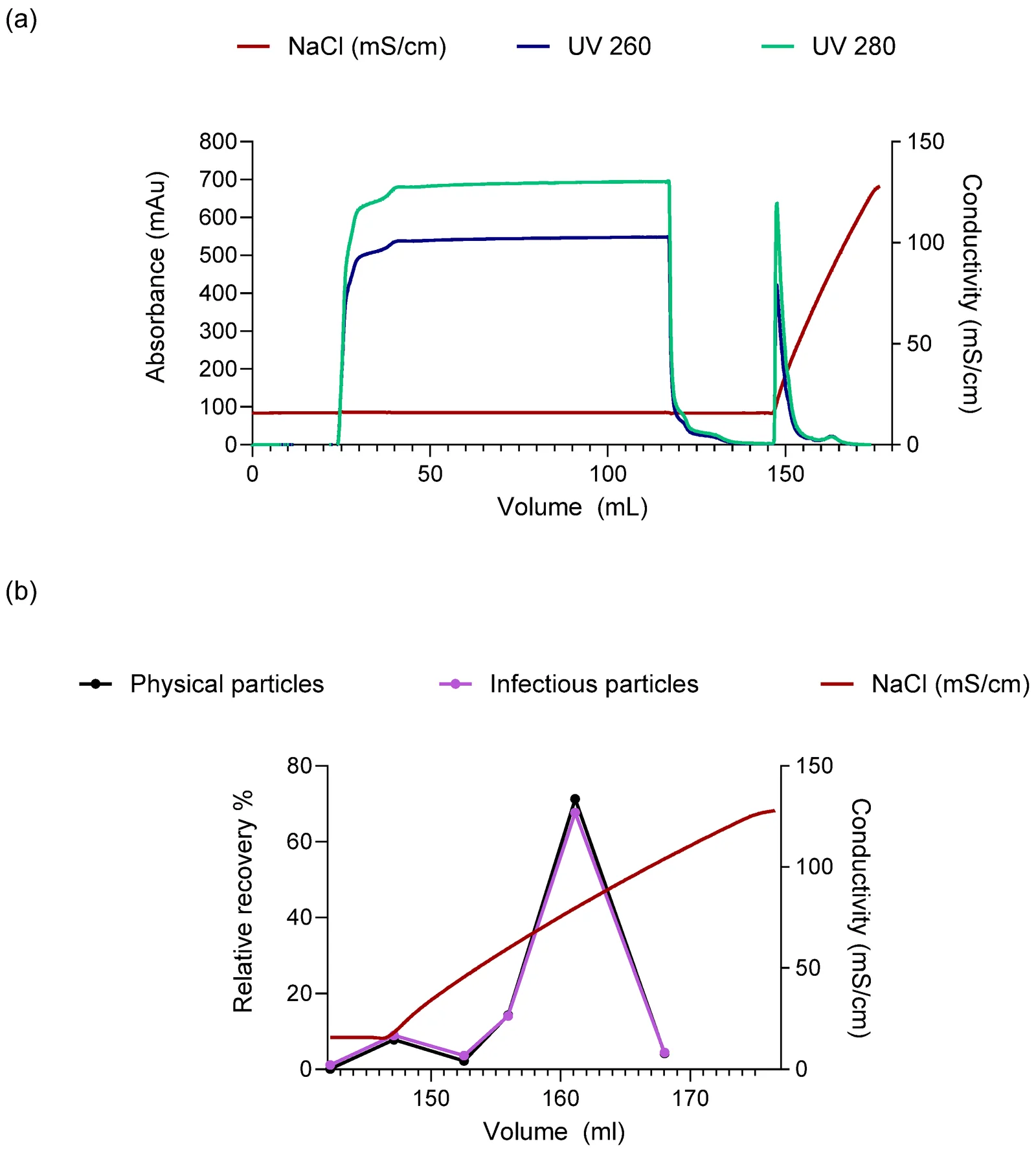
Results from AEX chromatography elution performed applying an increasing gradient of NaCl concentrations. (**a**) Chromatogram displaying the UV absorbance at 280 nm and 260 nm and the conductivity profile over the course of the elution volume. (**b**) Relative infectious and physical titers of the collected fractions are displayed along with the conductivity gradient and eluted volume.

One of the main steps of a typical DSP platform for LVV manufacture is the anionic exchange (AEX) chromatography. This method separates the negatively charged LVV particles from neutral and positively charged impurities. Specifically, during this study, AEX chromatography was performed using a membrane functionalized with quaternary ammonium (Q) ligands. The elution from Q membrane is performed by increasing salt concentration [20]. To identify the specific conductivity at which viral particles are detached, an elution conditions screening was carried out using a NaCl gradient from 0.15 M to 1.5 M, and fractions were collected across the conductivity range (Figure 5a). The results show a viral particle elution profile characterized by a predominant particle population released at high conductivity (Figure 5b). This pattern contrasts with the usual two distinct particle populations observed in transient transfection-based viral vector salt gradient elution profiles [21]. Specifically, 71.3% of the physical titer and 67.7% of the infectious titer recovered from the AEX chromatography were eluted at a conductivity of 79–104 mS/cm. This elution pattern allows to increase the washing salt concentration, which potentially enables more efficient clearance of impurities along an optimized AEX purification step.

Subsequently, we performed a complete DSP workflow following our industrial platform. Based on earlier findings showing that viral particles eluted at higher conductivity than typically observed, the preceding wash steps were adjusted from standard 0.3 M NaCl containing buffer to 0.4 M NaCl concentration to enhance impurity removal. The AEX chromatography step was performed with a membrane loading of 19,003.47 ng per mL of membrane volume and yielded a 42.77% recovery based on infectious titer measurements and 78.28% recovery regarding physical titer. All filtration steps throughout purification and concentration of the viral supernatant achieved recoveries above 80%. Overall, the DSP process from AEX chromatography onward yielded a 26.92% recovery of infectious titer and 59.05% of physical titer. Importantly, impurities associated with the viral suspension were effectively removed, achieving 99.11% DNA clearance and 98.56% HCP removal.

### 3.2. Genomic Analysis and Integration Method Assessment

#### 3.2.1. Whole Genome Sequencing (WGS) of the PCL

Our inducible LVV packaging cell line (PCL) contains integrated copies of the three helper genes required for lentiviral vector production (*Rev*, *VSV-G* and *Gag-Pol*) as well as the *tTA* transactivator. These helpers were randomly integrated into the cell genome and were previously analyzed by Targeted Locus Amplification (TLA) study to identify the integration loci and by qPCR to determine the integrated copy number [6]. Additionally, we performed a WGS analysis to characterize the integration site and structure of the integrated helper genes.

**Figure 6:**
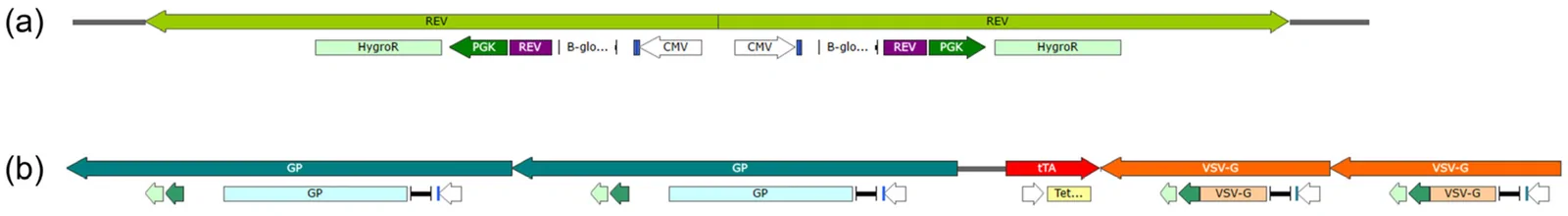
Schematic representation of the helper gene disposition of the integrations identified in the PCL by WGS. (**a**) Insertion containing *Rev* genes. (**b**) Insertion containing *Gag-Pol*, *tTA* and *VSV-G* genes.

As confirmed by the previous TLA, the WGS showed that two *Rev* copies were arranged in a head-to-head orientation on chromosome 6 (Figure 6a). In addition, we detected a large concatemer, likely resulting from the co-transfection of multiple constructs (Figure 6b). This concatemer includes full-length *tTA* insertion. On the left side of this element, we observed a complete *Gag-Pol* copy, present in 2 tandem repeats, according to multiple sequencing reads. On the right side, the *tTA* sequence is followed by a *VSV-G* copy in a confronted orientation, which also appears as 2 tandem repeats. Additionally, TLA data suggested that this multi-copy insertion is located on chromosome 3.

#### 3.2.2. Transposition-Mediated Integration for SCL Generation

As proof of concept throughout platform development, preliminary SCLs were generated by random transgene integration approach through transfection of linear DNA molecules into the reference PCL. During this process, linear DNA molecules may form concatemeric structures and integrate into the genome as tandem arrangements [22,23]. Additionally, linear DNA can be susceptible to exonuclease-mediated degradation, which may result in partial copy integration [24]. It does not represent a limitation for the helper genes, as the presence of intact copies was confirmed in the PCL. Nevertheless, the sequence and structural integrity of the therapeutic transgene copies must be strictly preserved since it will ultimately be packaged into the viral particle. In this context, transposase-mediated integration emerges as an alternative method for integration that offers a higher control of the integration event. The transposase of choice was SB100X, a hyperactive version of sleeping beauty with high transposition efficiency [25].

**Figure 7:**
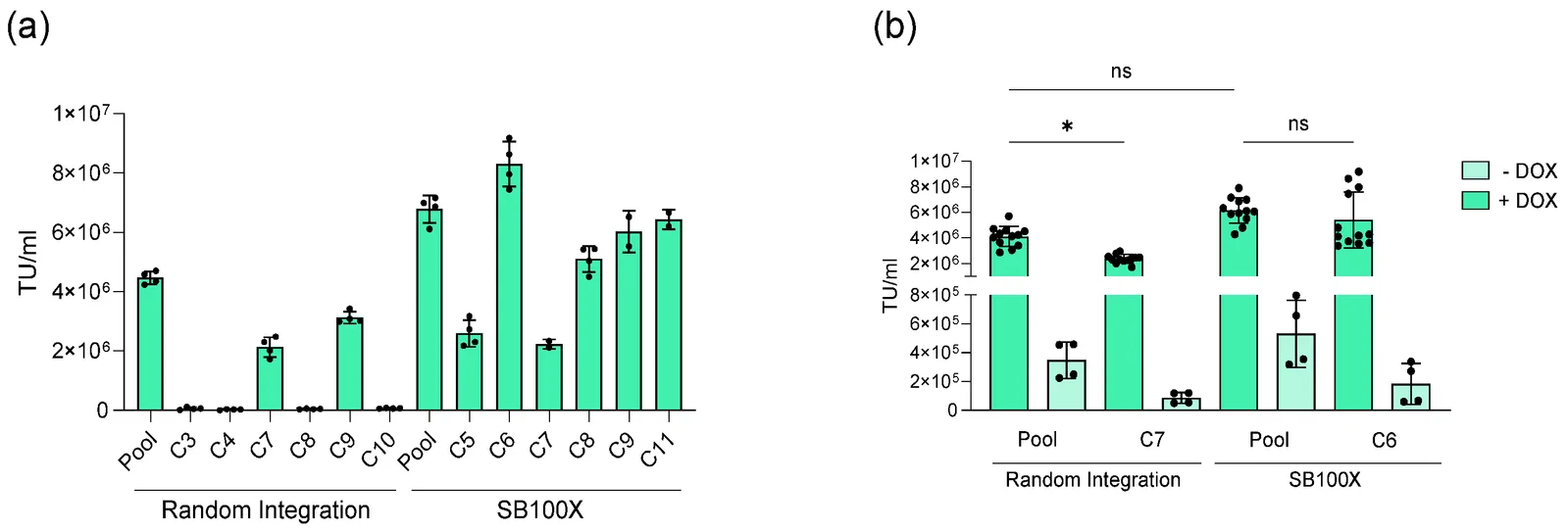
Clone screening study of SCL generated by random integration or SB100X-mediated integration of the transgene. Productive capacity of polyclonal and monoclonal SCL in TU/mL in presence and absence of doxycycline: (**a**) initial screening and (**b**) best performing clones. Data are expressed as mean values, with error bars representing standard deviations, and with dots representing individual technical replicates. Data from Figure 6b were subjected to Kruskal–Wallis followed by Dunnett’s multiple comparison test to compare the clones to the respective pool. * (*p* < 0.05).

A comparative analysis was conducted in which two SCL were generated by integrating the transgene either via random integration (SCL-RI) or via SB100X transposition (SCL-SB). Initial polyclonal cell populations showed similar infectious viral particle productivity between the two conditions. Nevertheless, to enable a more precise evaluation of integration events, the cell lines were followingly subcloned to obtain monoclonal cell lines. An initial screening study was performed to evaluate the productivity of these monoclonal derivatives in terms of LVV infectious titer. The results indicate an overall homogeneity in SCL-SB clones, while a higher variability between SCL-RI ones (Figure 7a). Nonetheless, the highest titer achieved by the top-performing clones were comparable to those of their respective parental cell lines, with SCL-CAR RI clone 9 reaching (3.13 ± 0.19) × 10^6^ TU/mL and SCL-CAR SB clone 6 reaching (8.04 ± 0.75) × 10^6^ TU/mL. Based on productivity results of the screening experiment along with the growth rate during culture, one clone from each condition was selected for further analysis, specifically clone 7 from RI clones and clone 6 from SB clones. Clones were again tested for LVV productivity, additionally evaluating the repression of the cell line production in the absence of doxycycline. The results show similar production capacity of both parental cells and their respective clones and an overall reduction in basal LVV production of the clones in the absence of doxycycline (Figure 7b).

**Stability study:**

The stability of SCL-CAR SB clone 6 and SCL-CAR RI clone 7 was assessed in terms of productivity and integrated transgene copy number quantification, as well as the occurrence of viral entry. For that, cells were aged during an estimated duplication number required for GMP-grade LVV manufacturing, covering cell expansion for banking and production processes.

**Figure 8:**
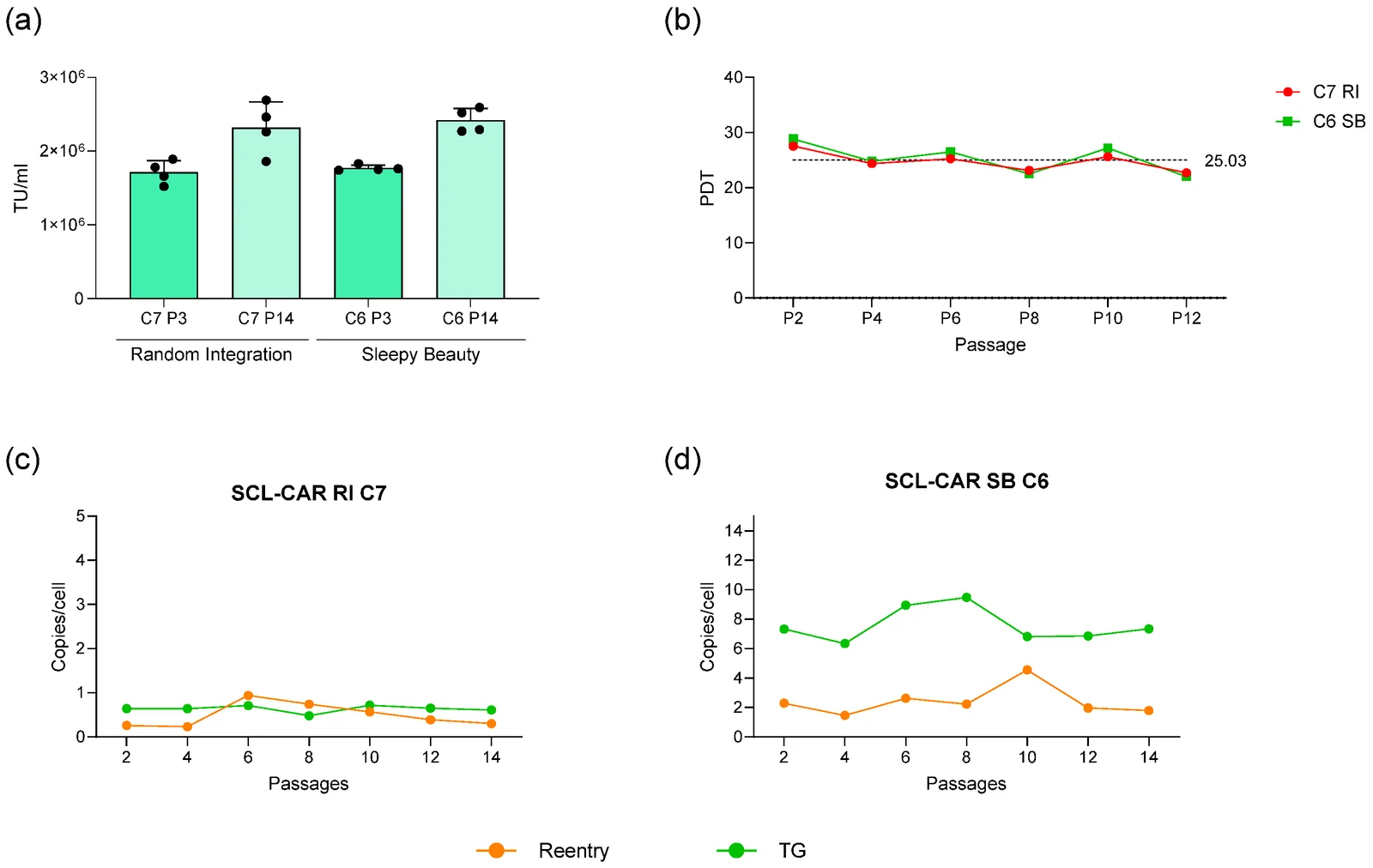
Stability study performed for SCL-CAR RI clone 7 and SCL-CAR SB clone 6. (**a**) Productive capacity of both clones in TU/mL at passage 3 and passage 14. Data are expressed as mean values, with error bars representing standard deviations, and with dots representing individual technical replicates. (**b**) Growth rate of both clones as population doubling time (PDT). Transgene copy number and produced LVV reentry are displayed in (**c**) for SCL-CAR RI clone 7 and (**d**) for SCL-CAR SB clone 6.

The results show that the productive capacity of both clones was stably maintained throughout the time in culture (Figure 8a). Their growth rate remained stable and similar between them, as the population doubling time was consistent for both clones throughout the study, with a mean PDT of 25.03 (Figure 8b). Lastly, qPCR analyses were performed to determine transgene copy number and LVV re-entry. The total number of integrated copies was measured by amplifying a cassette-specific region, while re-entry frequency was assessed by amplifying a region specific to retro-transcribed genomes. Transgene copy number was calculated by subtracting the re-entry value from the total copy number. The results show that SCL-CAR RI clone 7 contained one copy of the transgene and one virus re-entry per cell (Figure 8c). As for SCL-CAR SB clone 6, around eight copies of the transgene were integrated into the genome, and two viruses per cell appeared to reenter (Figure 8d). Overall, the number of transgene copies and re-entered LVV was stably maintained during culture of both clones.

**Targeted Locus Amplification (TLA):**

Previously, the PCL helper integrations were characterized by a TLA analysis [6] and an additional WGS that provided insight into integration copy number and their potential genomic locations. These analyses indicated that random integration efficiently inserted the constructs into the genome, although co-transfected genes frequently integrated at the same locus, forming concatemers or tandem arrangements. To further investigate the integration pattern of SB100X-mediated transposition and compare it with random integration, we carried out a TLA analysis of the transgene integration in SCL-CAR SB clone 6.

The results show high coverage across the vector with no evidence of plasmid backbone presence and identified 14 transgene integration events in different genomic locations (Figure S3). Among them, 10 represented transposase-mediated integrations, mapping to chromosomes 1, 2, 3, 5 (two copies), 7, 15 (two copies), 18 and X. One integration in chromosome 8 was consistent with lentiviral insertion, as the flanking sequences corresponded to LTR elements. The remaining three integration events were not successfully identified. Two integrations with one single genome-vector breakpoint identified were mapped next to the integrations in chromosomes 3 and 7. Due to the intrinsic limitations of TLA, whether these latter events represented a single complex insertion site or multiple independent sites remains undetermined, but were likely related to complex integrations. Nonetheless, promoter presence was not observed in these aberrant copies, thus representing no risk for potential truncated protein encoding or lentiviral vector packaging. An additional genome-vector breakpoint was identified in chromosome 8 that started with an ITR element, suggesting transposase-mediated integration. Nevertheless, the other breakpoint sequence was not identified due to the complex nature of the genomic sequence in this region. Compared with previous TLA analysis, the most notable difference relative to random integration was the absence of any vector–vector junctions, indicating that no concatemerized or tandem arrangements of co-transfected plasmids were present. This suggests that SB100X-mediated insertions occurred at distinct loci, with a single copy integrated at each site. These results are broadly consistent with the qPCR data previously obtained for transgene copy number quantification.

## 4. Discussion

Driven by the growing demand for reproducible and cost-efficient LVV production processes, one of the tendencies adopted by the manufacturing companies and research groups is the development of stable producer cell lines (SCLs) for LVV production [6,7,8,26]. These cell lines have integrated into their genome all the genetic material required for LVV manufacturing; thus, the induction of their expression is sufficient to provide consistent LVV yields [6].

As previously described [6], our team developed an SCL generation platform based on a well-characterized and stable lentiviral packaging cell line (PCL). As proof-of-concept, an SCL with a GFP-CAR-expressing transgene was generated to emulate a therapeutic context. Following regulatory guidelines, this cell line was subcloned for a monoclonal SCL-CAR obtention, which demonstrated genomic and functional stability, along with a potent repression of the inducible system, avoiding uncontrollable LVV production and genome instability due to potential retro-transduction [6]. All these preliminary studies were performed on a small-scale setup, which provided promising production levels required for basic research; nevertheless, substantially larger volumes of vector are required to support clinical and commercial stage-aiming manufacturing batches. Therefore, we conducted a scale-up study to assess the feasibility, and potential risks of implementing this system in a bioreactor setting, extending the process from a laboratory-scale condition to 2500-fold larger culture system.

The scale-up study demonstrated feasibility of SCL-based LVV manufacturing and showed that cell growth and metabolic profiles were comparable across scales, supporting the use of small-scale models as predictive tools for process development. Viral supernatant quality was maintained following scale-up, with similar infectivity and impurity profiles observed at both scales. However, despite these similarities, LVV productivity was lower at large scale, suggesting that one or more scale-dependent manufacturing parameters negatively impacted the overall process performance.

While large-scale systems usually maintain a stable and constant neutral pH during production, the small-scale condition tends to acidify due to cell growth. There is consistent scientific evidence supporting higher productivity associated with lower pH conditions [8,15,16,17]. Specifically, improved LVV yield titers observed under acidic pH conditions have been attributed, in part, to a reduction in retro-transduction of producer cells by newly synthesized LVVs, which has been reported to cause 70% vector yield loss [17,18,27]. Moreover, several studies indicate that acidic pH can promote a metabolic shift in which both lactate and glucose are simultaneously consumed, supporting enhanced cell growth and increased productivity [15,16,28]. Taken together, these effects offer a plausible explanation for the superior performance observed at a small scale, where pH-driven metabolic and process-related factors likely play a significant role. Notably, viral retro-transduction analysis in the scale-down experiment showed clear differences between batch-like and perfusion-like conditions. These differences in reentry frequency are attributed to the pH of the culture media, as VSV-G is well known to enter cells via the LDL receptor through a pH-dependent process [29,30]. Indeed, VSV-G suffers a reversible conformational change at slightly acidic pH, that avoids its interaction with LDLR [31], which turns out to be an efficient strategy to avoid retro-transduction [17].

Findings from the small- and large-scale experiments of this study offered valuable insight for refining bioreactor production conditions. On the one hand, cell health and metabolism are key determinants of cellular productive capacity, and results evidenced that, under the seeding density and culture conditions evaluated in this study, the optimal induction time point for this specific cell line is 2.5 g/L glucose concentration, which provides high productivity with low impurity levels. On the other hand, culture media acidification upon induction might be beneficial for enhanced LVV production. While LVV manufacturing has traditionally relied on neutral pH conditions, the use of perfusion systems reduces the time of viral particles in the culture supernatant and might therefore limit viral reentry. Nevertheless, our experimental data indicate that this effect alone is insufficient to prevent reentry. Notably, this data is supported by the literature, where slight pH shifts into 6.6–6.8 levels have demonstrated increased LVV yields [8,32]. However, the present study does not allow the definition of an optimal quantitative pH range for LVV production, which remains to be determined. Additionally, SCL productivity can be increased by extending harvesting time. Nonetheless, these preliminary findings require further validation through additional testing in bioreactor.

One of the most critical steps of industrial scale purification is the AEX chromatography due to the sensitivity of the LVV to shear stress and high salt concentrations [33,34]. This method is based on electrostatic binding of LVV to a charged membrane, followed by elution at high ionic strength. However, inconsistent performance is reported in the literature, with LVV losses typically ranging from 60 to 80% in large-scale procedures [35,36]. Generally, significant binding heterogeneity is observed in the LVV particles bound to the AEX column, which is characterized by a two-peak, salt-gradient elution profile, consisting of a weakly bound population (eluted with ∽400mM NaCl) and strongly bound population (eluted with ∽900mM NaCl) [37]. It remains unclear whether this binding heterogeneity arises from differences in proteoglycan distribution within the membrane, variations in viral envelope density or stochastic processes [21,33]. Nevertheless, there is strong evidence suggesting that LVV elution profiles reflect the presence of distinct LVV subpopulations driven by interparticle envelope heterogeneity [21]. In addition, transfection reagents such as PEI are known to electrostatically interact with the cell membrane, potentially altering membrane charge distribution and composition [38,39]. Thus, since the SCL-based LVV production platform relies on induction rather than transient transfection, we decided to perform a purification of SCL-derived LVV through AEX chromatography and analyze the elution profile under an increasing salt gradient. Contrasting with the usual elution profile, where two viral populations are recovered at different conductivities, the results show that these viruses eluted as a main single population. The elution occurred at conductivities between 80 and 105 mS/cm, which corresponds to the strongly bound population. These preliminary data suggest that SCL-derived LVVs exhibit reduced physicochemical heterogeneity and a shift toward a more negatively charged viral population. This observation may be associated with the elimination of transfection-related components, such as residual PEI, which could contribute to charge heterogeneity in transiently produced viral supernatants. However, additional studies are required to identify the specific particle attributes underlying the observed chromatographic profile. Nonetheless, the presence of a single viral particle population enabled the optimization of the washing steps, resulting in nearly complete impurity clearance and LVV recoveries consistent with values reported in the literature [35,36,40,41,42].

As stated earlier, our SCL generation platform relies on a stable, reproducible and extensively characterized packaging cell line (PCL). Because the genomic architecture of a production cell line can directly influence product quality, process performance and long-term stability, thorough genomic characterization is a critical component of cell line development. Regulatory agencies such as EMA and FDA expect producer cell lines used for Advanced Therapy Medicinal Products (ATMPs) to be thoroughly characterized with respect to identity, genetic stability, safety and suitability throughout the manufacturing process [43,44]. In this context, the advances in Next Generation Sequencing largely removed the technical barrier to obtaining whole-genome sequence information from engineered cell lines, but data interpretation remains a major challenge [45]. In many cases, the bottleneck is no longer sequencing itself, but rather the availability of appropriate reference genomes, accurate variant-calling pipelines and bioinformatic methods capable of resolving complex genomic rearrangements. Thus, the field still relies on scientifically justified analytical strategies to complement sequencing data. Consequently, orthogonal analytical approaches combining multiple complementary genomic and molecular assays represent an effective strategy for engineered cell line characterization.

In SCL development, integration strategies are essential for maintaining the structural integrity of the inserted genes. While random integration is generally attributed to non-homologous recombination events [22], co-transfection of multiple DNA constructs often results in concatemer formation, leading to the insertion of several copies at the same genomic locus [23,46]. Furthermore, the ends of linear DNA molecules during transfection are exposed to exonuclease-mediated degradation, which can lead to partial or incomplete gene integration [24]. Notably, we have found this approach to be highly effective, functional and stable for helper gene integration as demonstrated by the extensive functional and genomic characterization of the PCL [6]. However, transfer genome integration imposes more stringent requirements on sequence integrity, as it is ultimately packaged into viral particles and delivered to the patient; therefore, methods relying on linear DNA substrates may be less suitable despite their reported success [2,7,47]. In this context, transposition-mediated integration provides a more controlled integration strategy, preserving transgene integrity during genomic insertion. Although both methods can generate functional producer clones, SB100X enables higher integration efficiency, with demonstrated stability both genomically and functionally. Importantly, SB100X transposase was delivered as mRNA, restricting its activity to a transient period and minimizing the risk of transposon mobilization and unintended integration of the transposase sequence itself. In contrast, DNA-based transposase delivery strategies require evaluation of potential plasmid integration into the host genome, as reported in other stable producer cell line development approaches [48]. Furthermore, TLA analysis demonstrated that SB100X-mediated integration resulted in single-copy insertions at each integration locus, with no evidence of concatemer formation or tandem integration events. Together, these features support a well-characterized genetic architecture that facilitates clone selection and increases confidence in transgene integrity.

Although numerous LVV producer cell lines have been reported, meaningful comparisons of productivity and process performance across studies remain challenging, due to the intrinsic differences between platforms. These include the LVV transfer genome [11,49], envelope pseudotype [50,51,52] or adherent or suspension growth characteristics of the cells [7,8]. Furthermore, variations in manufacturing scale and process design, encompassing both upstream and downstream operations, can substantially influence LVV yield and quality. Cross-study comparisons are further complicated by differences in data reporting and by the use of different functional titration assays [53,54]. While direct benchmarking should therefore be interpreted with caution, comparison with established industrial LVV producer platforms provides useful context for evaluating the performance and characteristics of our SCL. Among these, some are based on suspension-adapted cells [47,48,55], whereas others remain adherent [2,56]. Suspension-based systems often achieve higher volumetric productivity because they can be cultivated at substantially greater cell densities. However, when compared with established adherent producer cell lines, our platform exhibits a comparable level of productivity [2,56], even though different vector designs and methods of SCL generation have been utilized. Furthermore, in contrast to our platform, these cell lines include the HIV-1 Trans-activator of Transcription (tat) protein, a regulatory protein that enhances LVV production, but is typically omitted from third-generation LVV systems to enhance the safety profile [1]. In addition to the competitive productivity of our SCL generation platform and the advantages of the transfer genome integration strategy employed, the cell line displayed long-term stability. This stability is in part provided by the inducible regulation of helper gene expression, which enables the selection of SCL clones with essentially no basal activity. This prevents uncontrolled vector generation and mitigates the risk of genome instability due to retro-transduction, thereby supporting the long-term stability of the producer cell line. Such stability is critical for maintaining a robust manufacturing platform and may represent an advantage over systems where long-term clone stability remains challenging [48].

Overall, the platform consists of an extensively characterized cell line for third-generation LVV production. It combines productive and genomic stability with a streamlined SCL generation workflow to provide a clinically relevant manufacturing platform that delivers efficient productivity within a simplified and potentially safer vector production system. Clone selection is guided by productivity, minimal basal expression, long-term stability and preservation of transgene integrity, enabling the rapid establishment of robust and well-characterized producer cell lines.

To sum up, stable producer cell lines offer multiple advantages in gene and cell therapies, largely driven by the improved cost efficiency of the manufacturing. Notably, the currently approved LVV-based therapies carry treatment costs ranging from around half to several million US dollars per patient. Among the various cost drivers, LVV manufacturing is considered a major contributor to the overall cost of the final therapeutic product [57]. Accordingly, improving manufacturing efficiency is critical to enhancing patient access and supporting the long-term sustainability of gene therapy commercialization. LVV manufacturing requires the development of reproducible, robust, and cost-effective production processes, and although current manufacturing methods are capable of supplying the market, continuous optimization is required to reduce process variability and production costs. These limitations support the implementation of stable producer cell line platforms as a next-generation manufacturing solution. The generation of stable cell lines for LVV manufacturing is a complex, labor-intensive and time-consuming process. However, advances in process automation, high-throughput subcloning platforms, efficient genome engineering technologies and next-generation sequencing have significantly accelerated cell line development. These innovations not only facilitate the efficient generation of stable producer cell lines but also provide powerful tools for their comprehensive functional and genomic characterization. As a result, the development of well-characterized, robust and scalable cell lines is becoming increasingly feasible, supporting the implementation of more reproducible and cost-efficient LVV manufacturing processes. Recently, the first CAR-T therapy utilizing stable cell line for LVV production earned FDA IND approval [58], which provides reasons for optimism. The present case study demonstrates the scalability of our SCL providing encouraging results, and identifies several opportunities for further optimization, offering valuable insights into key process parameters that could be refined to improve productivity and manufacturing robustness. The platform has undergone extensive genomic characterization, which provides a deeper understanding of the integration architecture and genetic stability of the cell line.

## Data Availability

The original contributions presented in this study are included in the article/Supplementary Material. Further inquiries can be directed to the corresponding author.

## Supplementary Materials

The following supporting information can be downloaded at: https://www.mdpi.com/article/10.3390/pharmaceutics18101265/s1, Figure S1: Glucose consumption and lactate production kinetics of both large- and small-scale cultures. Induction timings are marked with arrows. Large-scale production induced at (a) 2.5 g/L glucose and (b) 1.5 g/L glucose. (c) Small-scale glucose monitoring for both conditions. Glucose data for both small-scale conditions originate from a single parallel well; Figure S2: Comparison of LVV production under batch-like and perfusion-like conditions in a small-scale model. Producer cell viral re-entry analysis displayed as TU/cell in each day of production for conditions. Data are expressed as mean values, with error bars representing standard deviations; Figure S3: Targeted Locus Analysis schematic illustration of the transgene integrations in the chromosomes of the genome.

## Abbreviations

The following abbreviations are used in this manuscript:

AEX	Anionic Exchange
CDMO	Contract Development and Manufacturing Organization
DSP	Downstream Processing
GMP	Good Manufacturing Practice
HCP	Host Cell Protein
LVV	Lentiviral Vector
PCL	Packaging Cell Line
PDT	Population Doubling Time
PEI	Polyethyleneimine
SCL	Stable Cell Line
TLA	Targeted Locus Amplification
TU	Transduction Units
USP	Upstream Processing
VCN	Viral Copy Number
WGS	Whole Genome Sequencing
