# Supplementary material for: From Platform Development to Scale-Up: Stable Producer Cell Line-Based Lentiviral Vector Manufacturing
Source: Pharmaceutics. 2026 Oct 6;18(10):1265. doi: 10.3390/pharmaceutics18101265 (PMC13708141; doi:10.3390/pharmaceutics18101265)
Supplement: Supplementary file 1 [file pharmaceutics-18-01265-s001.zip › pharmaceutics-4497315-supplementary.pdf]

Supplementary Materials

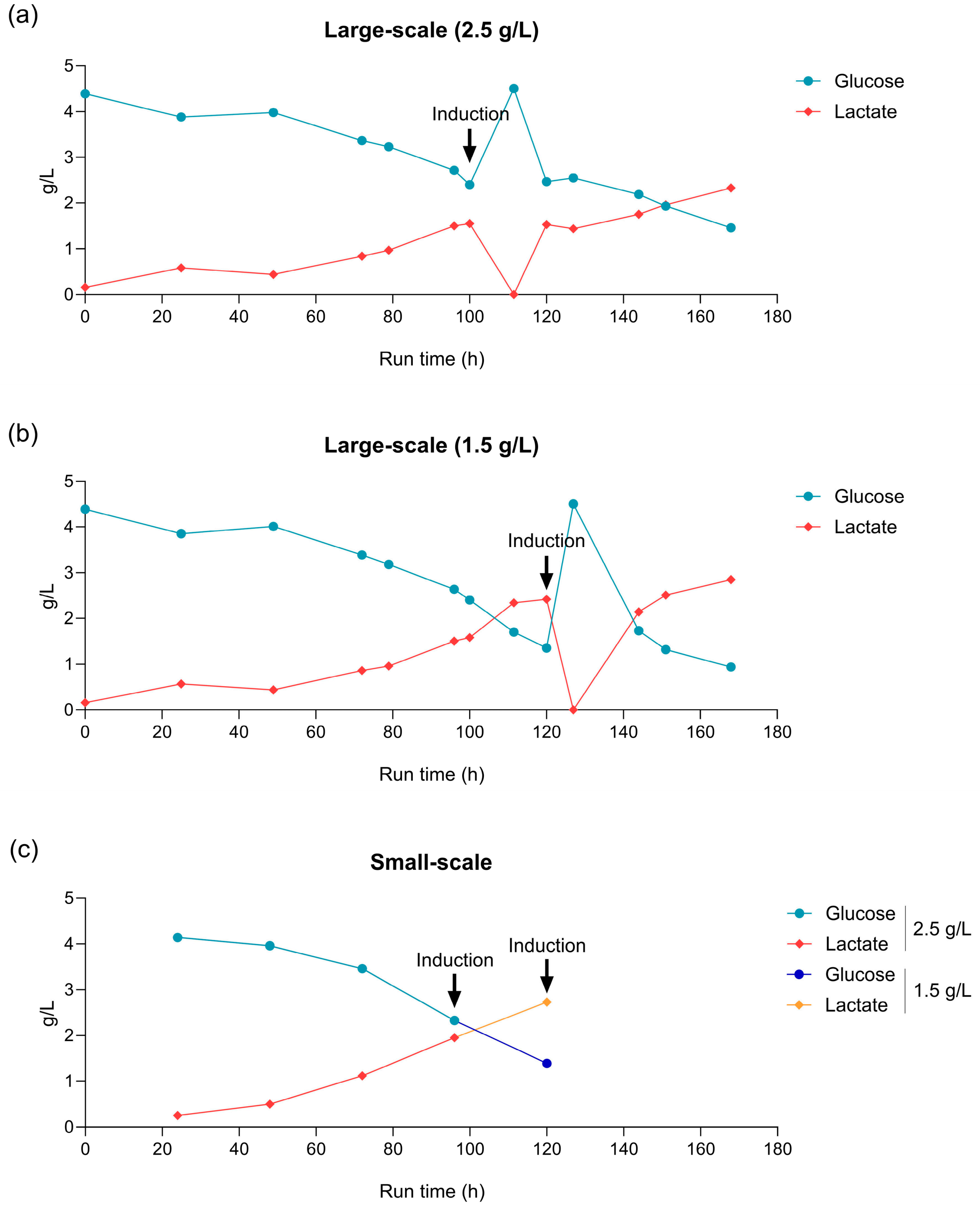

Figure S1: Glucose consumption and lactate production kinetics of both large- and small-scale cultures. Induction timings are marked with arrows. Large-scale production induced at (**a**) 2.5 g/L glucose and (**b**) 1.5 g/L glucose. (**c**) Small-scale glucose monitoring for both conditions. Glucose data for both small-scale conditions originate from a single parallel well.

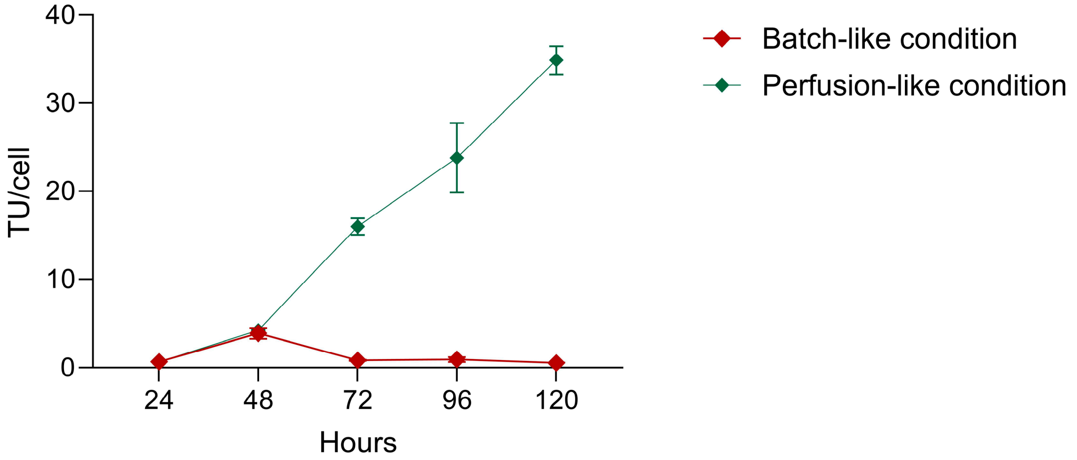

Figure S2: Comparison of LVV production under batch-like and perfusion-like conditions in a small-scale model. Producer cell viral re-entry analysis displayed as TU/cell in each day of production for conditions. Data are expressed as mean values, with error bars representing standard deviations.

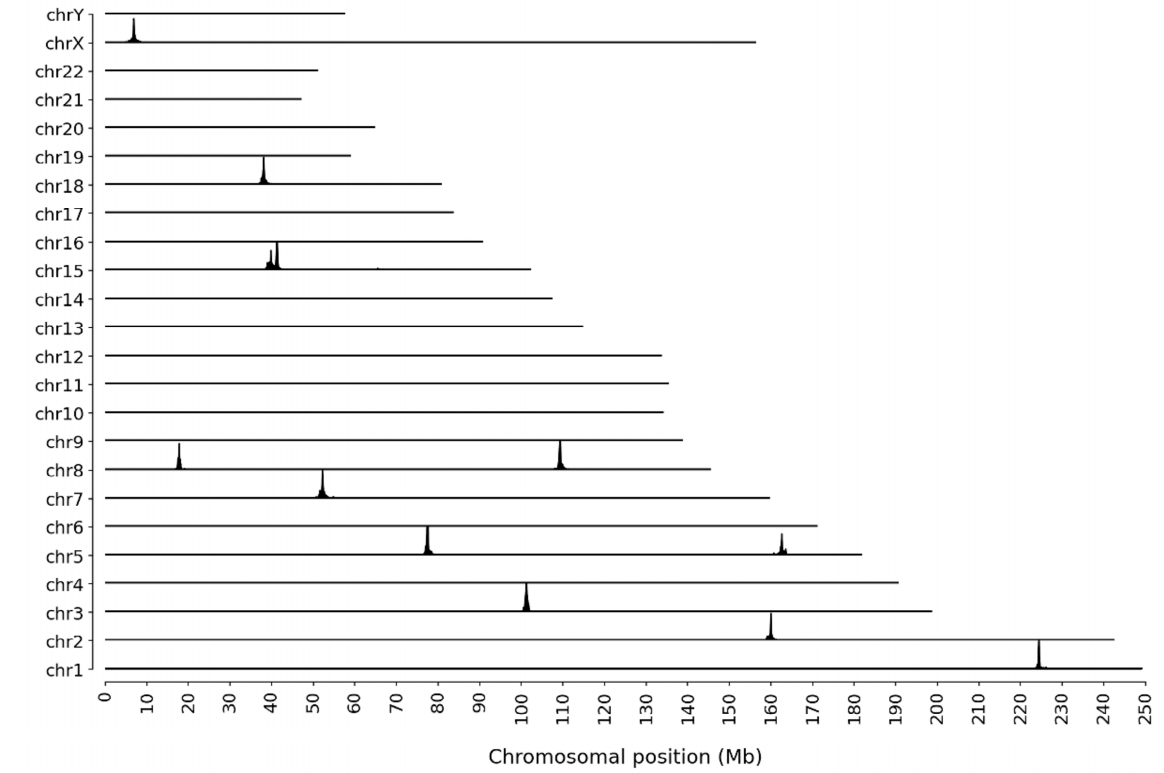

Figure S3: Targeted Locus Analysis schematic illustration of the transgene integrations in the chromosomes of the genome.
